# Traumatic brain injury, neuroimaging, and neurodegeneration

**DOI:** 10.3389/fnhum.2013.00395

**Published:** 2013-08-06

**Authors:** Erin D. Bigler

**Affiliations:** ^1^Department of Psychology, Brigham Young UniversityProvo, UT, USA; ^2^Neuroscience Center, Brigham Young UniversityProvo, UT, USA; ^3^Department of Psychiatry, University of UtahSalt Lake City, UT, USA; ^4^The Brain Institute of Utah, University of UtahSalt Lake City, UT, USA

**Keywords:** traumatic brain injury, TBI, brain development, neuroimaging, neurodegeneration, neuropsychiatric disorders

## Abstract

Depending on severity, traumatic brain injury (TBI) induces immediate neuropathological effects that in the mildest form may be transient but as severity increases results in neural damage and degeneration. The first phase of neural degeneration is explainable by the primary acute and secondary neuropathological effects initiated by the injury; however, neuroimaging studies demonstrate a prolonged period of pathological changes that progressively occur even during the chronic phase. This review examines how neuroimaging may be used in TBI to understand (1) the dynamic changes that occur in brain development relevant to understanding the effects of TBI and how these relate to developmental stage when the brain is injured, (2) how TBI interferes with age-typical brain development and the effects of aging thereafter, and (3) how TBI results in greater frontotemporolimbic damage, results in cerebral atrophy, and is more disruptive to white matter neural connectivity. Neuroimaging quantification in TBI demonstrates degenerative effects from brain injury over time. An adverse synergistic influence of TBI with aging may predispose the brain injured individual for the development of neuropsychiatric and neurodegenerative disorders long after surviving the brain injury.

Neuronal damage from traumatic brain injury (TBI) induces pathophysiological as well as anatomical changes (Blennow et al., 2012) that may set the stage that eventually leads to dementia (Shively et al., 2012). It is well-established and long-known that the damage from a TBI may be severe enough that the cognitive deficits experienced by the individual never return to pre-injury levels; thereby meeting *Diagnostic and Statistical Manual*—*Fourth Edition—Text Revision* (DSM-IV-TR) criteria for *Dementia Due to Head Trauma* (DSM-IV-TR 294.1x; see Bigler, 2007b, 2009). In the *DSM-5*, this is now classified as *Major Neurocognitive Disorder Due to TBI* (see American Psychiatric Association, 2013). However, accumulating evidence suggests with prior TBI, even in the individual that returns to presumed pre-injury cognitive ability that an increased risk for later in life degeneration occurs increasing the likelihood for a dementing illness (Plassman et al., 2000; Wang et al., 2012). It is this latter aspect of the long term effects of TBI on the aging process that will be the focus of this review. Since the majority of head injuries resulting in TBI occur before middle-age, the basic question examined in this review is the potential role that prior TBI plays in the aging process and the mechanisms whereby prior TBI would adversely influence aging.

When neural tissue is injured and reparative and restorative mechanisms fail to work, cellular morphology changes; this may ultimately result in cell death (Stoica and Faden, 2010). With change in cellular morphology or cell death, either regional or whole brain atrophy results, depending on the severity and type of injury (Pitkanen et al., 1998; Bramlett and Dietrich, 2007; Lifshitz et al., 2007; Tata and Anderson, 2010). In a human post-mortem TBI study of brain volume, Maxwell et al. (2010) examined brain weight of TBI patients who survived several months to years post-injury but were moderately to severely disabled or in a vegetative state. The following brain weights (± standard deviation) were reported: 1442.7 ± 105.0 g for controls, 1329.6 ± 202.9 g for moderately disabled, 1330 ± 140.7 g for severely disabled, and 1275 ± 135.5 g for vegetative state patients. On average moderate-to-severe TBI resulted in approximately a 112 cc of generalized volume loss at post mortem in these relatively young TBI patients (on average 44 years of age at the time of injury and 52 years at the time of death), when compared to age-matched controls who died from non-TBI related causes. While the Maxwell et al. sample was middle age at the time of death, as a group the amount of overall volume loss documented at post-mortem was comparable to that observed in patients 20–30 years older (mean age 71.1 ± 8.3) with various types of dementia at the time of death (see Purohit et al., 2011).

The Maxwell et al. study confirms TBI associated total brain volume (TBV) loss at post-mortem in the patient with chronic brain injury that approximates the degree of brain volume loss in those with brain atrophy from various types of age-related degenerative diseases much later in life. Fortunately, contemporary neuroimaging provides methods for ante-mortem detection of volume loss and its relationship to outcome following TBI, including the prediction of adverse neurological and neuropsychiatric outcome. Having sustained a TBI raises the potential for serious long-term neurobehavioral sequelae (Moretti et al., 2012). Since smaller TBV or brain volume loss from injury, disease or disorder may be a factor associated with a host of neurological and neuropsychiatric disorders (Kempton et al., 2008; Okonkwo et al., 2010; Olesen et al., 2011; Gunther et al., 2012; Skoog et al., 2012), if TBI reduces brain volume, such reductions likely relate to adverse outcome.

For the individual who sustains a TBI and survives the injury, the post-injured brain has to navigate the remainder of life with potentially less resiliency and reserve because of the parenchymal loss (Bigler, 2007b, 2009). Since aging alone—even healthy disease-free aging—is nonetheless associated with brain volume loss, does having TBI-related volume loss accelerate the loss associated with aging? Since less brain volume later in life increases the risk of dementia (Skoog et al., 2012) does brain volume loss from TBI relate to increased dementia and possibly induce further degenerative changes?

TBI induces a number of neuropathological changes like the aggregation of β-amyloid and tau along with neuroinflammatory changes that resemble the pathology of degenerative diseases (Blennow et al., 2012). Do the combination of effects that resemble later in life neurodegenerative changes in the young individual who sustains a TBI become associated with a greater likelihood for transition to a progressive dementing illness later in life?

These questions are addressed in this review which examines volume loss from brain injury, its role in the aging process, and how neuroimaging methods may be used to document such changes.

## TBI and parenchymal volume loss

### Significant TBI results in brain volume loss

**Figure 1:**
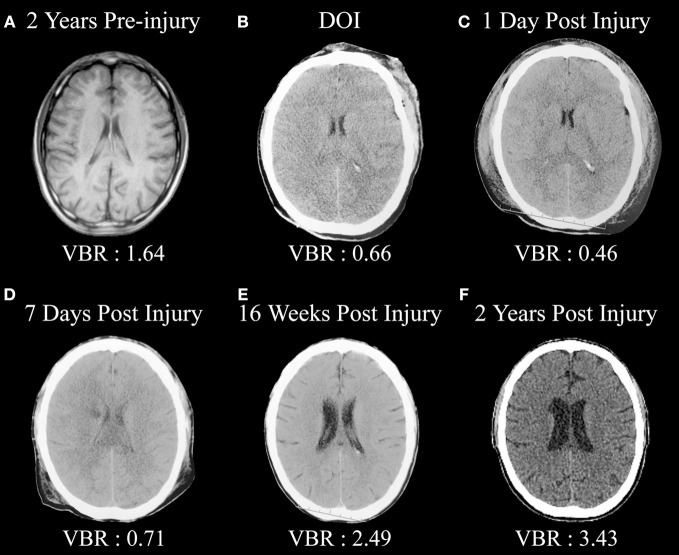
**(A)** Pre-injury magnetic resonance image (MRI) approximately 2 years prior to a severe traumatic brain injury (TBI). Note the normal size of the ventricular system and ventricle-to-brain (VBR) ratio of 1.64 (normal is approximately 1.5 with a 0.5 standard deviation). **(B)** Day-of-injury initial CT demonstrating brain edema and reduced VBR, which continues to be reflected in **(C,D)**. **(E)** Distinct neurodegeneration has occurred by 16 weeks post-injury, reflected as ventricular dilation and increased VBR, with continued neurodegeneration out to 2 years post-injury as seen in **(F)**.

Figure 1 is from an adult male who sustained a severe TBI (GCS = 3) but in whom a pre-injury magnetic resonance imaging (MRI) scan had been performed, so pre-injury quantification of ventricular and brain volume could be established. An excellent global measure of brain integrity is the ventricle-to-brain ratio (VBR; Bigler and Tate, 2001), calculated as the total of ventricular volume divided by total brain volume (TBV), multiplied by 100 so that the ratio is reported in whole numbers. Overall, normal VBR for the adult is around 1.5 with a 0.5 standard deviation (Blatter et al., 1995). In cerebral atrophy the reduction in brain volume is accompanied by a passive, compensatory increase in ventricular volume (referred to as hydrocephalus ex vacuo) thereby resulting in increased VBR. Acutely in TBI, presence of cerebral edema results in increased parenchymal volume because of tissue expansion from swelling combined with ventricular compression, reducing the size of the ventricle. In the mildest forms of TBI when edema occurs, this swelling may be transient and brain parenchyma and ventricular volume return to pre-injury levels. If injury is of sufficient severity, however, over time parenchymal degeneration occurs, reflected by brain volume loss in conjunction with hydrocephalus ex vacuo and increased VBR. As seen in this illustration, the pre-injury scan VBR was well within normal limits. However, post-injury computed tomography (CT) findings reflect distinctly reduced VBR, indicative of whole brain edema and ventricular compression, which remained throughout the first week post-injury. However, by 16 weeks post-injury substantial ventricular increase is evident along with a higher VBR score, which continued to increase over the next 2 years post-injury.

Over time, these scans, as shown in Figure 1, demonstrate several important points. Compared to the pre-injury brain, post-injury generalized swelling and ventricular compression in the acute and early sub-acute timeframe clearly indicate the non-specific edema and generalized neuroinflammation affects the entirety of the brain. Furthermore, this type of swelling likely compromises overall cerebral perfusion, thereby affecting neural integrity, cellular degradation and apoptosis (Xu et al., 2010) and in rodent TBI models exacerbates hippocampal damage beyond what occurs directly from the TBI (Foley et al., 2013). By the time of the first scan was obtained more than an hour post-injury in the case shown in Figure 1, obviously the instantaneous biomechanical shear/strain deformation injuries had occurred and what is primarily being viewed in the first acute scan are the combinations of primary and the initial secondary effects of the injury. By day 7, there is a low density lesion, likely an infarction beginning to evolve adjacent to the caudate, which is particularly evident 2 years post-injury. This demonstrates another TBI principle in that distinctly focal effects, either from shearing and/or vascular effects, may occur within the backdrop of global pathological changes. What is reflected in the scan 2 years post-injury is the summation of all of the pathological effects of TBI—mechanical deformation, axonal shearing resulting in primary axotomy, and likely focal lesion effects as well as the combined effects of secondary axotomy, ischemic damaged from compromised cerebral blood flow and whatever pathological neuroexcitatory effects may have occurred in combination with other neuroinflammatory reactions (Bigler and Maxwell, 2011, 2012). The pathological cascade is complex and as shown in this illustration plays out over an extended period of time. The end-product, however, is a brain reflective of non-specific damage with considerable overall TBV loss.

In a living veteran sample with penetrating brain injury and post-traumatic epilepsy, as a group TBI patients were found to exhibit approximately a 52 cc whole brain volume loss based on neuroimaging findings obtained years post-injury (Raymont et al., 2010). The TBI subjects in the Raymont et al. investigation had sustained injuries not as severe as in the subjects in the Maxwell et al. (2010) investigation and were penetrating in nature, but still exhibited substantial volume loss based on quantitative neuroimaging. Thus, when assessed with *in vivo* neuroimaging methods, TBI may result in substantial volume loss of brain parenchyma, which in turn relates to neurocognitive outcome (Tate et al., 2011), to be reviewed below. Furthermore, *in vivo* quantitative neuroimaging provides methods to examine the course of neurodegenerative changes over time post-injury in those who survive the brain injury.

If the trauma induced volume loss associated with TBI were just the effects of the initial injury, once the acute cascade of degeneration occurred it would be assumed that the injured brain should exhibit no further degeneration. However, if TBI has produced something more than a static brain injury, longitudinal neuroimaging would exhibit progressive changes over time (Greenberg et al., 2008). This would implicate neurodegenerative processes extending well-beyond the point of acute TBI, and the sub-acute timeframe necessary for those initial pathological effects to run their course (Bendlin et al., 2008; Ng et al., 2008; Farbota et al., 2012). Recently, chronic neuroinflammation, particularly involving white matter (WM) has been implicated in some of these progressive changes, including volume loss in the corpus callosum (Johnson et al., 2011, 2013). Since the shear-strain influences of TBI are more likely to damage axons, producing what is referred to as traumatic axonal injury (TAI; see Bigler and Maxwell, 2012) any chronic neuroinflammatory response influencing WM integrity would likely have adverse influences on recovery.

Longer-term pathological effects from TBI, regardless of their nature, would likely interact with the aging process and may set the stage for adverse neuropsychiatric and neurocognitive outcome after injury along with increased risk for age-related neurodegenerative diseases (Lucas et al., 2006; Johnson et al., 2012; Shively et al., 2012). Based on neuroimaging studies of normal brain development over the life span in comparison to the pathological effects of TBI, this review will address three basic issues: (1) dynamic changes in brain volume relate to age, with “normal” age-related reductions in brain volume occurring after the third decade in life, (2) TBI interferes with age-typical brain development depending on the age when injury occurs and while both gray matter (GM) and WM are damaged, trauma selectively damages axons; thereby more disruptive to WM neural connectivity during the aging process post-injury (see Ramlackhansingh et al., 2011), and (3) along with whole-brain, WM and GM volume reductions from TBI, traumatic injury results in more selective frontotemporolimbic damage, atrophic changes identifiable via neuroimaging. Diffuse damage, along with the frontotemporolimbic locus of damage from TBI, pre-disposes the brain injured individual for increased neuropsychiatric morbidity with aging and increased risk for dementia later in life.

## Dynamic changes in brain volume over the life span

**Figure 2:**
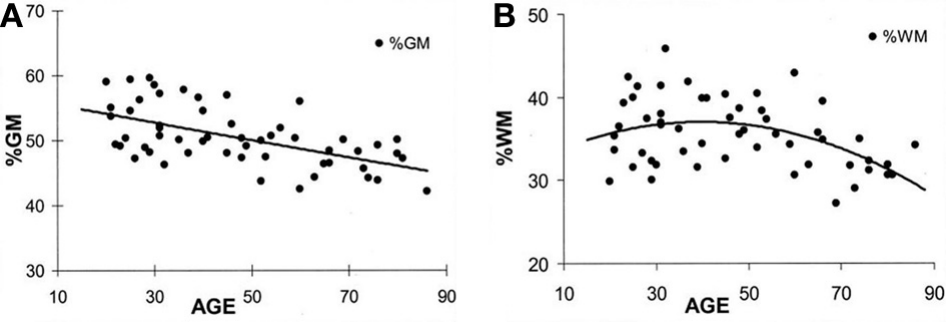
**(A,B) These plots are from Ge et al. (2002) and reflect different trajectories of white matter (WM) and gray matter (GM) over the life-span.** The percent of GM volume total brain (TBV) volume declines with age whereas the percentage of WM to TBV shows and increase followed by decline later in life. Note that the age at which an individual is injured occurs not in a static brain, but rather in a brain that has age-dependent changes occurring simultaneously with the age when injury occurs. Reproduced with permission from The American Society of Neuroradiology.

**Figure 3:**
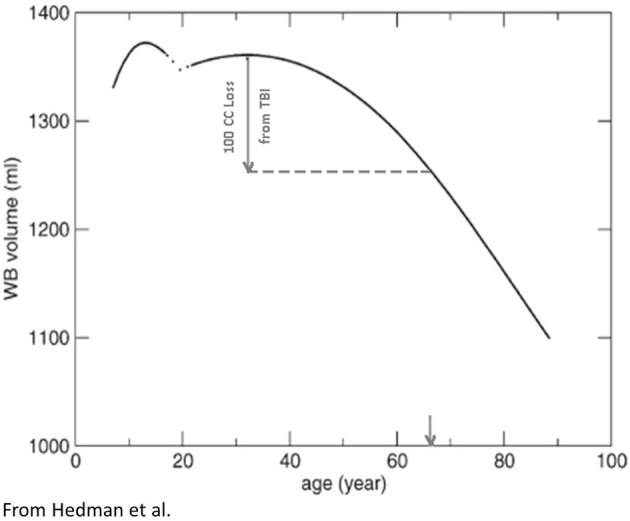
**Based on a meta-analysis Hedman et al. (2011) constructed the following TBV plots over the life span from approximately age 4 through 90 years of age.** A hypothetical TBI patient injured in their 20's sustaining a volume loss of a 100 cc is depicted with the inference being that although only chronologically a young adult, because of the brain loss, the total reduction of TBV is similar to someone in their 7th decade of life (downward arrow, X-axis). In other words, purely from a TBV perspective, TBI accelerated brain volume loss. Reproduced with permission from Wiley.

At birth, TBV is approximately 25% of what it will become in adulthood but the volume increase in brain growth occurs rapidly, where based on magnetic resonance imaging (MRI) volumetric studies by 8 years of age, TBV approximates adulthood (Courchesne et al., 2000). Over the remaining childhood years and throughout adolescence, a dynamic interaction between cellular maturation and pruning along with myelination results in reduced overall GM with increased WM (see Figure 2). Knowing the influence of age-typical effects on brain, WM and GM volumes and the fact that all three are in decline later in the aging process (>6th decade of life) indicates their utility as neuroimaging markers of brain parenchymal health earlier in life. Quantitative neuroimaging methods that measure volumetric brain changes demonstrate these effects as shown in Figure 2. For example, Ge et al. (2002) plotted the percentage of whole brain WM and GM volume by age from approximately 10 through 90 years, as shown in Figure 2. Note that by mid-childhood GM has already started to decrease, which in childhood is thought to reflect normal neuronal pruning, all-the-while WM from late childhood through early adulthood increases and does not peak until early mid-adulthood but thereafter in decline just as GM. Throughout childhood and adolescence TBV reflects several dynamic phases of pruning, modeling and myelination but following peak development in adulthood thereafter a steady decline in TBV occurs as shown in Figure 3, adapted from Hedman et al. (2011). The Hedman et al. investigation examined 56 longitudinal MRI studies involving 2211 subjects from four to 88 years of age where they determined that after 35 years of age, a 0.2% per annum volume loss occurred which accelerated to 0.5% per annum after age 60.

Therefore, the early dynamic interplay between GM pruning and increased WM connectivity underlies much of the early ebb-and-flow of overall brain volume within the first three decades of life (see Figures 2, 3). However, after stabilization around mid-adulthood, brain volume follows an inexorable decline, which is age dependent. In the developing healthy brain, maturational changes may be measured as volumetric changes, including WM volume; WM connectivity also can be measured with metrics such as fractional anisotropy (FA) based on diffusion tensor imaging (DTI; Shi et al., 2012), MRI structural covariance functions (Zielinski et al., 2010) and functional MRI (fMRI; Rubia, 2012).

Since intracranial volume peaks in mid-childhood, around 8 years of age, and basically remains invariant for the remainder of life (Courchesne et al., 2000), any reduction in TBV is met with increased cerebrospinal fluid (CSF) volume, where notably increasing age results in a linear CSF increase (Inglese and Ge, 2004). Increased whole brain CSF with aging, disease or injury is a reflection of cerebral atrophy (Driscoll et al., 2011). When a significant TBI occurs with resulting volume loss from the injury that injury occurs amidst a developmental backdrop of changing TBV, ventricular and total CSF volume, WM and GM volumes at the time of injury (Tasker, 2006).

Prospective, life-span neuroimaging studies on the effects of TBI have not been done but inferences can be made from longitudinal and childhood developmental studies that have examined TBI patients in the chronic phase post-injury. In child TBI the injury perturbs developmental trajectories which may never return to their pre-injury trajectories (Tasker, 2006; Ewing-Cobbs et al., 2008; Wu et al., 2010; Beauchamp et al., 2011). In adults, any volume loss from brain injury is superimposed on whatever the age-typical volume loss would be, potentially resulting in an acceleration of any age-mediated decline (Bigler, 2007b, 2009). For example, returning to Figure 3, if a typical 25-year-old with a pre-injury TBV of 1350 cc (average adult brain volume) lost 100 cc because of a severe TBI (thereby approximating the volume loss for moderately-to-severely disabled individuals with TBI from the Maxwell et al. (2010) study mentioned above) as determined by quantitative neuroimaging several months post-injury that 25-year-old individual would have a TBV equivalent to a 65-year-old (note the point of intercept in Figure 3 and the down-pointing arrow). Did the brain injury with a 100 cc volume loss impose a 40+ aging effect on the brain in what should be a 25-year-old brain?

Figures 2, 3 are straightforward volumetric markers of brain development, and while they reflect gross anatomy, there are other neuroimaging biomarkers of both WM and GM integrity more sensitive to microstructure and neuronal health that also map onto these volume changes. Such changes show age-related dynamic alterations in energy metabolism including magnetic resonance spectroscopy findings (MRS), magnetization transfer ratios that directly assess WM integrity along with DTI, and resting state fMRI or rs-fMRI (Inglese and Ge, 2004; Rosazza and Minati, 2011). DTI and rs-fMRI findings in normal development and TBI are particularly important because these neuroimaging tools provide techniques for more directly assessing brain connectivity (Van Den Heuvel and Sporns, 2011; Irimia et al., 2012b). How brain connectivity is either maintained, adapted to or damaged is key to understanding the effects of TBI at any point in the life span as well as normal aging and neurodegenerative disorders (Liu et al., 2013; Steffener et al., 2012; Levin and Smith, 2013; Pandit et al., 2013). Fortunately overall parenchymal volume positively correlates with DTI metrics, especially WM volume (see Harrison et al., 2011, 2013) and thereby TBV and WM volumes likely represent proxies that reflect brain connectivity. Based on the age-dependent volume changes shown in Figures 2, 3, volume measures may be used as biomarkers of underlying brain health, developmental stage and brain connectivity. Reductions in brain volume from TBI would reflect reduced brain connectivity (Palacios et al., 2012, 2013).

**Figure 4:**
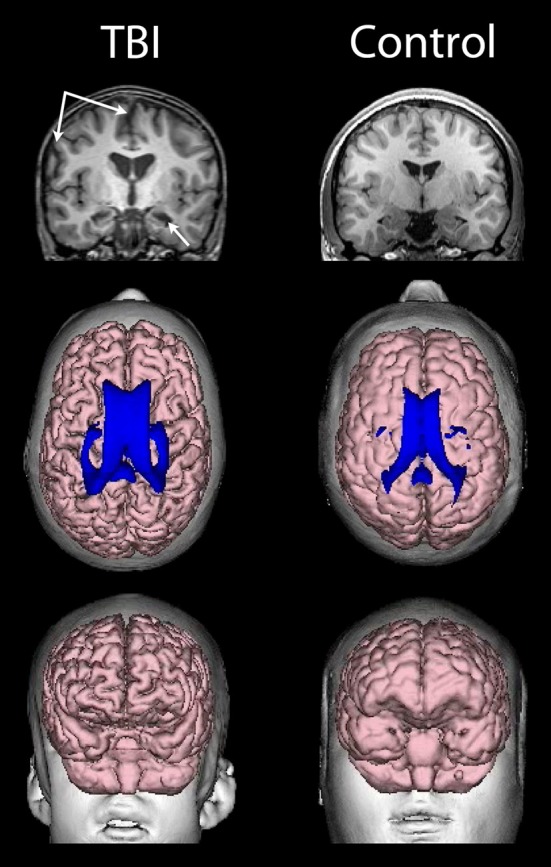
**(Top) T1-weighted coronal images of a TBI patient on the left and an age-matched control on the right, both young adolescent males.** Upper left-hand arrows point to a prominent interhemispheric fissure and cortical sulci, reflective of whole brain volume loss and generalized cerebral atrophy in the TBI patient. The lower arrow points to an atrophic hippocampus and dilated temporal horn, bilaterally in the TBI patient. **(Middle)** Dorsal view of a 3-D reconstruction of the ventricle in shown in blue superimposed on the flesh-tone brain surface 3-D reconstruction. Calculating whole brain volume and dividing it by total ventricular volume and multiplying by 100 results in a ventricle-to-brain ratio (VBR) of 5.55, which is markedly deviant from normal, which in typical developing controls is generally in the range of 1.5 with a 0.5 standard deviation [see Blatter et al. (1995) and Chang et al. (2005)]. The control subject VBR was 1.45. Frontal view of the 3-D reconstructed brain **(Bottom)** of the individual with TBI showing global frontal atrophy with visibly larger cortical sulci compared to the age-match control subject on the right, again reflective of generalized cerebral atrophy. Increased VBR reflects this type of global brain volume loss, ventricular enlargement, gyral shrinkage and sulcal enlargement.

As already introduced, the VBR metric represents a simple neuroimaging measurement sensitive to brain parenchymal volume loss as well as changes in CSF that relates to cognitive outcome in TBI (Tate et al., 2011). As rendered from a volume acquisition T1-weighted MRI, Figure 4 depicts a three-dimensional surface appearance of the brain along with the cerebral ventricles (in blue) in a healthy control compared to a TBI patient with severe brain injury, global cerebral atrophy (note the prominence of the cortical sulci and inter-hemispheric fissure) and increased VBR. What is important about this image of the brain injured patient shown in Figure 4 is that the TBI occurred when this individual was 12 years of age and the MRI obtained approximately 2 years post-injury. So the distinctly visible atrophic brain is that of a 14-year-old, but the degree of atrophy is similar to an individual seven or eight decades older. The advantage of using the VBR metric is that it automatically adjusts for head size differences due to height, body type and sex differences that influence head and brain size (Lainhart et al., 2006). In typical developing individuals, because normal brain development fills the cranial vault and ventricular size is minimal, VBR findings during childhood are relatively constant after age six and remain so throughout childhood, adolescence and early adulthood. Increases in VBR do occur in normal aging that become overtly notable by middle age but sharply increase after age 65. Chronic VBR changes reflective of generalized atrophy in TBI are directly proportional to the severity of injury (Bigler et al., 2006; Wilde et al., 2006a; Ghosh et al., 2009). Likewise, pathological increases in VBR are found in neurodegenerative disorders (Bigler et al., 2004; Carmichael et al., 2007; Olesen et al., 2011). The VBR in this child was calculated to be 5.55, which in comparison to a “normal” aging VBR would not have occurred until after the 8th decade of life. What will become of this brain as it ages?

## Brain volume reductions in TBI

Presumably, as injury severity increases more numerous and potentially widely distributed pathological effects occur throughout the brain (Adams et al., 2011). This fact likely characterizes the association between injury severity and reduced TBV as reflected by increased VBR. Regardless of how severity is defined (GCS, LOC or PTA) increased severity is associated with increased cerebral atrophy (Bigler et al., 2006), where VBR may triple or more in those with the most severe injury.

Examining VBR changes at different time points post-injury provides insight into the more long-term neurodegenerative effects from sustaining a brain injury. Blatter et al. (1997) examined in a cross-sectional sample VBR at different times post-injury showing dynamic atrophic changes with VBR increases more than 2 years post-injury. The steepest VBR increases post-injury occurred by approximately 3 weeks clearly reflecting the initial neuropathological effects of neuronal death and cellular phagocytosis that results in reduced TBV. However, VBR changes continued to increase in this study beyond 2 years after injury.

**Figure 5:**
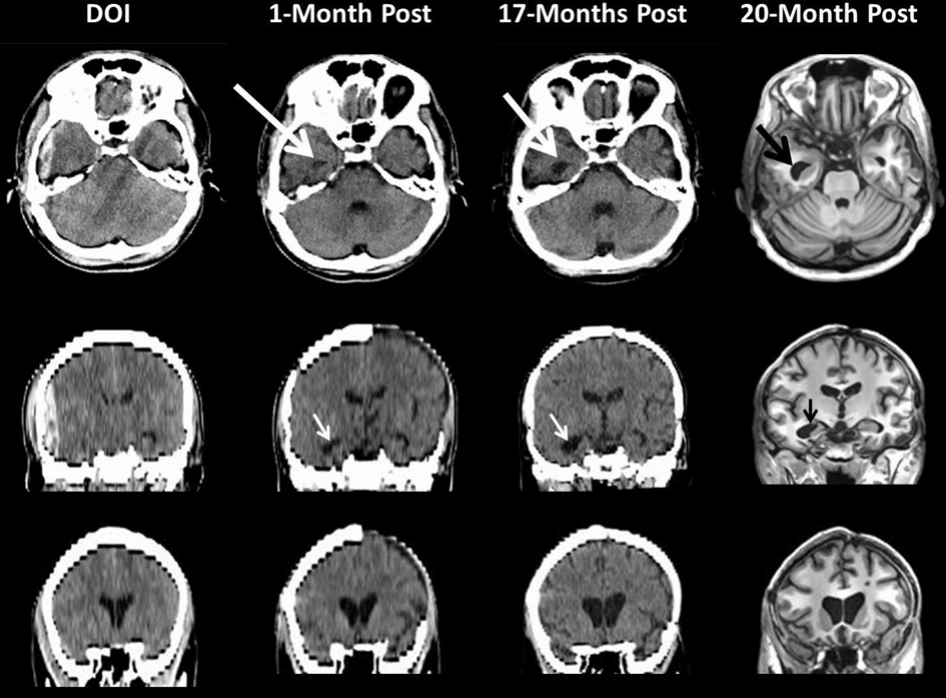
**This patient sustained a severe TBI as a consequence of a fall.** Note on the day of injury (DOI) the computed tomography (CT) scan demonstrates the presence of a large epidural hematoma with brain displacement. Repeat scanning was performed at 1-month (CT scan), 17 (CT scan) and 20 months (MRI) post-injury. For the CT scans in the middle and bottom rows the coronal sections shown are based on re-sampled axial images with degradation in image resolution but sufficient to depict ventricular changes over time. Note how in the DOI scan the temporal horn is basically undetectable from parenchymal shift from the epidural as well as edema but clearly visible and dilated by 1-Month (white arrow) which increases by 17-months and even more prominent by 20 months as shown in the MRI findings. The bottom coronal images clearly depict increasing dilation of the anterior horns of the lateral ventricular system reflecting brain parenchymal volume loss that progresses from DOI through 20-months post-injury. Note at 1 month the patient still has missing bone-flap from the original craniotomy to treat a contra coup hemorrhagic contusion and subdural hematoma.

Once trauma passes a pathological threshold, *in vitro* studies show that the primary trauma-induced cell loss begins immediately or within hours of injury depending on mechanism of injury, injury severity and the type of induced pathological effects (Cullen et al., 2011). However, as discussed by Bigler and Maxwell (2012) there are any number of potential secondary pathological pathways that could result in more long-term neurodegenerative effects. Confirmation of these long-term effects comes from other investigations as well. For example, Ng et al. (2008) first quantified CSF volume at approximately 4.5 months post-injury, long past the initial sub-acute time frame where Blatter et al. and others (see also Gale et al., 1995) have shown the greatest degree of degenerative change occurs from TBI, continued to show volumetric differences out to 2.5 years post-injury. In severe TBI these visible changes are readily viewed in the individual patient by sequential neuroimaging studies as shown in Figure 5. This patient sustained a severe TBI and while significant neurodegenerative effects had occurred by one month post-injury, visible changes progressed over the next 20 months based on scan findings.

In terms of actual volume loss, Sidaros et al. (2009) examined a group of severe TBI patients at approximately 8 weeks post-injury and then again at 12 months. In comparison to controls TBV was reduced by approximately 8.4% within this initial 2-month post-injury timeframe; however, using the patient's 2-month post-acute MRI as the baseline, by 12 months post-injury an additional overall 4% volume loss occurred (range from −0.6 to −9.4%). Translating this into actual brain parenchymal tissue loss (refer to Figure 3 again), in the typical 1350 cc brain, moderate-to-severe TBI would result on average in more than a 100 cc loss of brain parenchyma. Accordingly, the example given in Figure 3 actually does reflect a type of reduction in TBV that would occur in the typical young adult sustaining a severe TBI.

From the above discussion of brain volume loss, returning to the Hedman et al. (2011) investigation, given their report of a 0.2–0.5% per annum whole brain volume loss, if severe TBI results in a ~10% volume reduction this far exceeds any per annum “normal” volume loss. In fact that would induce a volume loss that in normal aging would have taken decades to achieve. In adulthood, depending on the age at the time of injury, such a volume loss likely adds to the aging burden on the brain and may accelerate age-effects by several decades. Although overly simplistic, the argument can be made that this volume loss is registered against whatever brain reserve capacity might have been present at the time of the original TBI (Bigler, 2007b), predisposing the individual with TBI to age-mediated neuropsychiatric and neurodegenerative disorders (Bigler, 2009). Traumatic-induced TBV reductions that occur within the first few months post-injury likely occur as a direct effect of the initial pathological response but more long-term TBV reductions would also reflect potential complex interactive age, neuroinflammation and neurodegenerative effects (Amor et al., 2010; Johnson et al., 2013).

**Figure 6:**
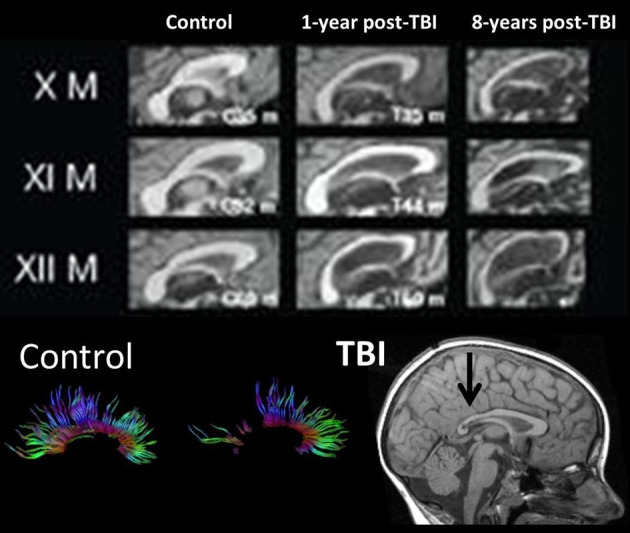
**(TOP) Mid-sagittal section through the corpus callosum showing initial atrophy 1-year from TBI, but increasing atrophy within this WM structure expressed over the next 8 years, indicating late neurodegenerative effects on WM.** Images reproduced with permission from Tomaiuolo et al. (2012) and Elsevier Science. **(BOTTOM)** Corpus callosum tractography extracted from DTI in a control, compared to a child with severe TBI. The mid-sagittal MRI shows gross thinning of the posterior corpus callosum (dark arrow) but DTI actually demonstrates that this reduced area actually has regions of no DTI-identifiable aggregate WM tracts. Adapted from Wilde et al. (2006b) used with permission from Mary Ann Liebert Publishing.

In the longest follow-up TBI study to date involving structural neuroimaging, Tomaiuolo et al. (2012) compared patients at one and then 8 years post-injury. Figure 6 from that study shows progressive changes in the corpus callosum, clearly implicating that further WM degeneration occurs long after the initial active pathological changes within the acute and sub-acute timeframe. Within a year post-injury, as also clearly visible in Figure 6, the corpus callosum goes through an initial loss of tracts and overall significant size reduction compared to an age-matched non-TBI control. For comparison, Figure 6, at the bottom, also shows normal appearance of DTI-derived fiber tracts involving the corpus callosum and the distinct loss of tracts that may occur in TBI taken from the study by Wilde et al. (2006a). The initial changes within the corpus callosum would mostly be attributable to the acute/sub-acute neuropathological effects including cell death and Wallerian degeneration of WM tracts. However, as shown in the Tomaiuolo et al. study, the continuation of WM degenerative changes from one out to eight years post-injury—as reflected in the continued reduction of the corpus callosum—could not be explained by the initial acute/sub-acute effects and implicates more long-term neurodegenerative sequelae. Tomaiuolo et al. also examined the volume of the hippocampus which interestingly, although significantly smaller than controls in the TBI subjects, did not show additional volume loss out to 8 years post-injury. Such findings are consistent with the progressive yet selective damaging effects of TBI on WM, including long-term neurodegenerative effects which will be discussed next.

The vulnerability of the corpus callosum in TBI is of particular interest, because some of the greatest shear/strain effects from trauma occur within the corpus callosum (McAllister et al., 2012; Rowson et al., 2012) and reductions in the size and/or integrity of the corpus callosum, even in mild TBI is well-documented (Aoki et al., 2012). The Tomaiuolo et al. (2012) study demonstrates that whatever initial traumatic effects there are on WM, damage to corpus callosum tracts may progress long after the injury, reflected as corpus callosum volume loss and atrophic changes (see also Tasker, 2006). Furthermore, Galanaud et al. (2012) show that pathological findings on DTI 1 year post-injury predict poor outcome from severe TBI. Interestingly, as already mentioned, Tomaiuolo et al. did not observe progressive changes in the hippocampus. It is very likely that different regions will have different resiliencies and/or vulnerabilities to the effects of injury and aging.

For example, progressive changes in the corpus callosum, long after injury, implicate active degenerative effects that are probably more than just age-mediated degenerative changes specific to WM (Farbota et al., 2012). Given the sensitivity of the DTI technique to detect abnormalities of myelin integrity and gliosis from TBI (Budde et al., 2011; Budde and Frank, 2012), DTI studies of abnormal WM in TBI should be able to document progressive degenerative changes in inter- and intra-hemispheric pathways (see Kim et al., 2008) when prospectively done.

## White matter vulnerability, diminished brain connectivity of TBI and changes over time

While hemispheric interconnectivity occurs across the corpus callosum, large intra-hemispheric fasciculi connect multiple regions within each hemisphere and likewise, there are long-coursing WM tracts integrating the brainstem and cerebellum with subcortical nuclei and the cerebrum. Shorter U-fibers connect adjacent gyral regions. The previously mentioned study by Bendlin et al., 2008 examined TBI patients (GCS of 13 or below) at 2 months post-injury and followed for more than 1 year post-injury showing longitudinal differences occurred not just within the corpus callosum, but also involving long white matter tracts as well as more short U-fibers. Follow-up with this same cohort out to 4 years post-injury has shown that the degeneration continues years post injury (Farbota et al., 2012). Wang et al. (2011) examined TBI patients earlier in their course of injury (from day-of-injury to within 9 days) and as with Bendlin et al. they followed-up within 14 months post injury and likewise documented ongoing degeneration. Thus, progressive WM degradation has now been documented that extends well into the chronic phase of having sustained a TBI (see also Sidaros et al., 2008, 2009) and Palacios et al. (2012, 2013).

Probably the most salient clinical effect of WM vulnerability is the loss of overall brain connectivity that occurs with TBI (Bonnelle et al., 2012; Caeyenberghs et al., 2012; Irimia et al., 2012a; Palacios et al., 2012). Caeyenberghs et al. (2012) not only showed the presence of disrupted WM integrity in TBI but that this disruption results in diminished cognitive control in those with brain injury. Furthermore, Wang et al. (2011) demonstrated that WM abnormalities both acutely and chronically were predictive of outcome.

**Figure 7:**
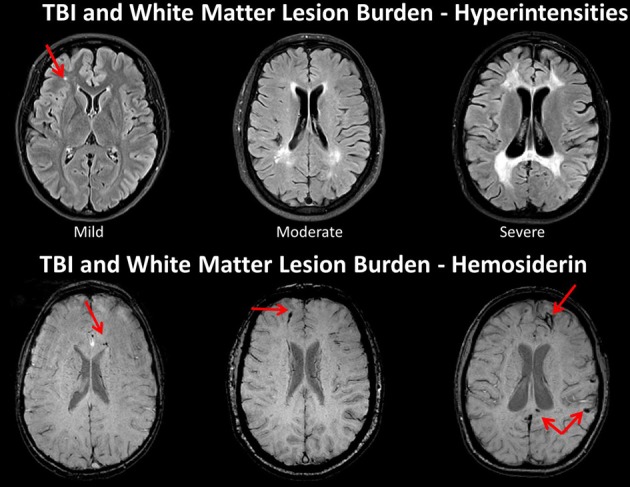
**TOP: Fluid attenuated inversion recovery (FLAIR) sequence in three traumatic brain injury (TBI) cases depicting different levels of white matter burden.** (Left) a child with mild TBI (mTBI) indicating a solitary, focal white matter hyperintensity (WMH). (Middle) a 62-year-old male with a severe TBI with no white matter abnormalities noted on admission CT. Patient had a GCS of 7 prior to intubation, meeting criteria for severe TBI (Right) 17-year-old injured 2 years prior with an admission GCS of 3. Note the prominent and extensive WMHs widely distributed. **BOTTOM**: The middle and right hand subjects are the same as above, but subject on the left side is a different child with a mild TBI, who did not have a WMH, but did show hemosiderin in the corpus callosum (arrow). Note that both patients with severe injury have some generalized atrophy and ventricular dilation as a reflection of generalized brain volume loss as a consequence of severe TBI along with multiple hemosiderin deposits.

The degree of WM integrity inferred directly or indirectly has been the focus of numerous studies in aging and dementia. Given improvements in image quantification the degree of WM lesion burden in the elderly individual is associated with increased levels of dementia and in those where changes in WM are quantified over time, the degree of WM burden predicts transition from mild cognitive impairment (MCI) to Alzheimer's disease (Carlson et al., 2008; Price et al., 2012; Silbert et al., 2012). Silbert et al. (2012), in a longitudinal, prospective neuroimaging study that measured WM volume as well as CSF, identified WM changes 10 years prior to MCI onset. Silbert et al. concluded that “acceleration in WM burden, a common indicator of cerebrovascular disease in the elderly, is a pathological change that emerges early in the presymptomatic phase leading to MCI (p. 741).” In the Silbert et al. investigation, they began neuroimaging studies at age 70, but if the change from 70 to 80 predicts who converts to MCI, what does this mean if baseline WM burden has already been compromised in a TBI patient injured at a much younger age? Figure 7 shows WM burden in terms of the fluid attenuated inversion recovery or FLAIR signal abnormality and susceptibility weighted imaging (SWI) sequences in TBI patients only one of whom is an adult.

## Frontotemporolimbic locus of damage from TBI

McAllister (2011) reviews the neuropathological as well as neuroimaging studies that demonstrate a frontotemporolimbic locus of injury from TBI. Anatomically, Bigler (2007a, 2008) has shown that because of the location of the cranial fossa and dura mater, trauma induced movement of the frontal and temporal lobes creates a mechanical vulnerability for damage to frontotemporolimbic regions of the brain following trauma. Brain deformations involving these structures, in turn increases the likelihood for focal pathology including surface contusions in these regions. Additionally, because of a unique confluence of major WM fasciculi that course through key brain regions in conjunction with network hubs and nodes involving frontotemporolimbic circuitry, even small but strategic WM lesions may have dramatic effects in TBI (Bigler et al., 2010).

The hippocampus has long been recognized as a fundamental limbic system structure. Hippocampal atrophy is a common finding in TBI (Bigler and Maxwell, 2011). As shown in Figures 3, 4, marked bilateral hippocampal atrophy may occur in severe TBI. The selective damage to the hippocampus in TBI is in part related to its positioning within the medial temporal lobe and the above mentioned biomechanical vulnerability of the medial temporal lobe to compression injury, but there are also intrinsic excitotoxic, neurotransmitter and metabolic factors specific to the hippocampus that predispose it to injury as well (see Diaz-Arrastia et al., 2009). Controlled animal experiments show that the hippocampus is particularly sensitive to the effects of trauma, even in mild TBI (Chen et al., 2012), with progressive neuronal death and hippocampal atrophy beyond the acute/sub-acute timeframe (Smith et al., 1997; Immonen et al., 2009). Additionally, given its high metabolic demands combined with over-expression of excitotoxic effects, greater hippocampal damage in TBI is often the outcome (Deng and Xu, 2011; Marquez de la Plata et al., 2011). In children, Wilde et al. (2006b) have shown that hippocampal volume loss from TBI was proportionally the greatest in comparison to all other brain regions examined.

Hippocampal and medial temporal lobe pathology plays a role in many neuropsychiatric and neurodegenerative disorders. For example, obvious pathological changes within the hippocampal formation occur in Alzheimer's disease and related dementias, associated with the memory impairments observed in these disorders (Hodges, 2012). Some level of hippocampal pathology is thought to contribute to major depression that occurs late in life, which also may relate to mild cognitive impairment (MCI; Morra et al., 2009). Additionally, a very complex interplay exists between TBV, aging and hippocampal volume and the transitions from healthy aging to MCI, and from MCI to frank dementia (Apostolova et al., 2012). Successful “aging” of the hippocampal complex and its multimodal efferent and afferent connections is considered a key element of brain plasticity with advancing age (Goh and Park, 2009). Oppositely, any injury to the hippocampus or its afferent/efferent connections such as from TBI likely adds to or advances the aging burden.

Lastly, when the limbic system is viewed in its entirety, it is a complex, highly interconnected system dependent on the integrity of numerous WM pathways. While the hippocampus is central to limbic system integrity, note that functional hippocampal disruption in TBI may occur by lesions quite distal to the hippocampus but occurring within other structures or limbic pathways that either input or output the hippocampus. For example, Wilde et al. (2010) have shown the vulnerability of frontal projections from the anterior cingulum in TBI. Such pathological changes would be a minimum of three synaptic connections from the hippocampus, and while the axonal injury may be specific to the cingulum bundle, it potentially would have some similar effects as if the damage actually had occurred in the hippocampus since part of hippocampal output to frontal cortex projects via the anterior cingulum. More directly, the fornix is vulnerable in TBI (Yallampalli et al., 2013), thereby disrupting the direct output of the hippocampus. Because of the interdependence of the limbic network on each of its constituent parts, intactness at one level does not insure that transfer of information occurs at another. Because of the increased likelihood of hippocampal damage in TBI, even minimal hippocampal damage could be highly disruptive to limbic connectivity and overtime, add to the burden of age effects.

## Aging, TBI and neurodegenerative disorders

**Figure 8:**
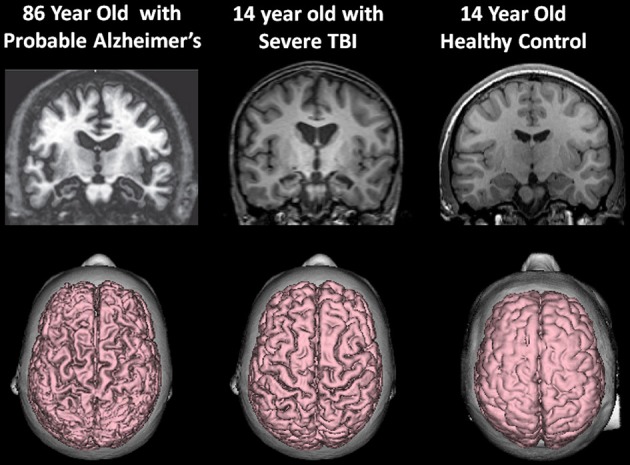
**(Top) T1-weighted coronal images approximately at the same level showing hippocampal atrophy in the 86-year-old patient with a clinical diagnosis of Alzheimer's disease and the 14-year-old patient with severe traumatic brain injury (TBI).** Note compared to the control coronal image on the right, both the TBI case and the Alzheimer's exhibit hippocampal atrophy, ventricular dilation and sulcal widening. **(Bottom)** All images are 3-D renderings from volume acquisition magnetic resonance imaging (MRI) depicting the dorsal view of the brain in each subject described above. Note the similarity of the diffuse pattern of atrophic change that has occurred in both the patient with Alzheimer's disease and the adolescent who survived severe TBI. Clearly, the elderly patient with Alzheimer's has more severe atrophy but nonetheless the atrophy in the TBI adolescent is substantial, especially when compared to the typical developing adolescent control. Note: The patient with Alzheimer's disease is taken from Jacobson et al. (2009); this patient's clinical findings, including additional neuroimaging and neuropsychological details are described in that publication. Reproduced with permission from John Wiley and Sons.

At the beginning of this discussion it was shown that whole-brain volume is age-dependent, which over time, even in the healthy individual declines. Brain atrophy outside the parameters of that related to normal aging is associated with a host of disease processes and potential adverse age-mediated genetic factors. As such, any environmental condition, such as a TBI that has the potential to influence brain volume loss likely adds to the disease burden that accompanies reduced brain volume during the normal aging process. This is graphically depicted in Figure 8 when the 14-year-old atrophic brain from a severe TBI is compared side-by-side with the MRI findings from an 86-year-old patient with Alzheimer's disease.

The initial, direct effect of brain injury induces neuroinflammatory reactions that may set the stage for long-term neuroinflammatory effects and neurodegeneration (see Ramlackhansingh et al., 2011; Johnson et al., 2013). The initial direct neurodegenerative changes that occur following TBI are manifested by significant brain atrophy that occurs within the first 6 months post-injury (Gale et al., 1995). While these effects can be accounted for by acute/sub-acute injury mechanisms and their pathological consequences, given the above observations, neuroinflammatory and neurodegenerative processes may extend for years beyond the initial post-injury injury time frame in the TBI patient. These more chronic effects do set the stage for important interactions that occur between the age at the time of injury, aging and age-related vulnerabilities, to later in life neuropsychiatric and progressive neurodegenerative disorders indicating that the lesion in TBI may be much more dynamic (Bigler, 2013). The frontotemporolimbic locus of where TBI induced degenerative changes are most likely to occur in the brain, has a most interesting overlap with brain areas observed in older individuals with increased risk for a variety of age-related neurological and neuropsychiatric disorders. Two examples are given that demonstrate this overlap. Sperling et al. (2011) review the pre-clinical stages of Alzheimer's disease in relationship to key biomarkers that, overtime, increase the likelihood for developing dementia.

As in the Jack et al. (2013) review, each of the biomarkers associated with the development of Alzheimer's disease, is also associated with biomarkers relevant to TBI; in particular, the deposition of the amyloid-beta (Aβ), neuroimaging based findings of pathology and presence of cognitive impairment. Aβ, tau pathologies, reduced brain volume, and impaired memory are all part of the pathological and clinical picture of TBI *and* age-associated degenerative diseases (Smith et al., 1999; Emmerling et al., 2000; Ikonomovic et al., 2004; Dekosky et al., 2010), creating the potential association between TBI and Alzheimer's disease and related dementias (Sivanandam and Thakur, 2012). In the Jack et al. review, two individuals are shown at the identical time-point—one with low risk for developing dementia, the other with high risk, for example, a prior history of TBI. Given the associations reviewed above, if the high risk individual were someone with a significant TBI who has recovered to some pre-injury baseline, yet had increased burden because of brain atrophy, mildly reduced cognitive ability and various biomarkers of neuronal injury, given this model the TBI individual would be at higher risk for developing dementia.

**Figure 9:**
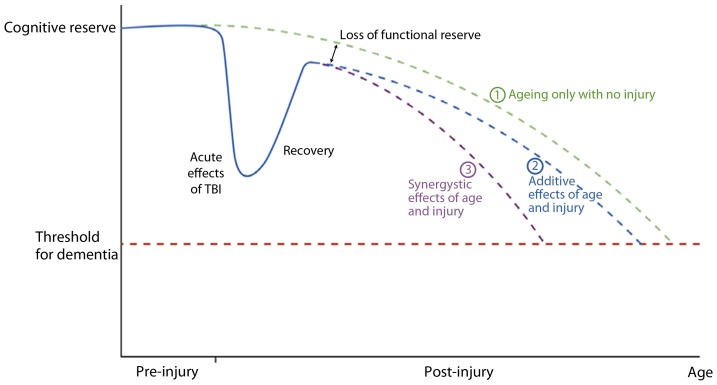
**A graphical representation of a postulated cognitive reserve and how head injury may increase the risk of cognitive decline.** The broken green line (1) represents the “normal” situation. There is loss of cognitive function with aging until a threshold point is crossed (broken red line) resulting clinically in dementia. After an episode of traumatic brain injury there is a significant decline in cognitive function which recovers, the degree of recovery being dependent on the severity of the head injury. Recovery is, however, not complete resulting in a loss of functional reserve. After this point cognitive decline may be as for normal ageing [broken blue line (2)] with the dementia threshold being crossed earlier due to loss of functional reserve, or there may be a continued synergistic effect of mechanisms initiated by the head injury which accelerates cognitive decline [broken purple line (3)]. Reproduced with permission from John Wiley and Sons and Smith (2013).

Smith (2013) reviewed the long-term consequences of microglial activation associated with TBI and summarized the potential findings as shown in Figure 9. This diagram uses the cognitive reserve hypothesis (see Bigler, 2007b) that assumes an inexorable yet normal decline in function with age. Although the brain adapts when injured except in cases of severe catastrophic injuries, the individual may never return to baseline and then as depicted in the illustration the adverse effects may be either synergistic or additive. Regardless of the mechanism, having a brain injury shortens the time for when the dementia threshold would be achieved.

A second example comes from the review by Bozzali et al. (2008) on regional and global atrophy that accompanies cognitive decline in degenerative disease, its frontotemporolimbic distribution and a more direct link between having sustained TBI earlier in life and developing a dementing illness (Plassman et al., 2000; Magnoni and Brody, 2010; Esiri and Chance, 2012; Sivanandam and Thakur, 2012; Wang et al., 2012). The implication from these studies and reviews is that overlapping trauma-induced neurodegenerative effects occur within the same frontotemporolimbic areas associated with age-related neurodegenerative disorders. This association between TBI and later onset of dementia may also relate to the selective WM damage that occurs with TBI and the role that WM pathology plays in the expression of dementia via a breakdown in neural connectivity and networks (Carmichael and Salloway, 2012; Shively et al., 2012; Weinstein et al., 2013).

The above discussion focused mostly on a single event, moderate-to-severe TBI, but since mild TBI (mTBI) constitutes the majority of injuries, if mTBI contributes adversely to the aging process, this could be a major public health concern (Bazarian et al., 2009). Similarly, multiple concussions or mTBIs are commonplace in some sports (Harmon et al., 2013). Chronic traumatic encephalopathy (CTE) has been demonstrated in athletes with repetitive blows to the head (Victoroff, 2013), yet not necessarily meeting criteria for a clinically diagnosed concussion (McKee et al., 2012). In the largest post-mortem study to date of 85 individuals with CTE, McKee and colleagues have shown that CTE, while it may in some cases be the solitary neuropathological diagnosis, CTE was also associated with cases of Alzheimer's disease, Lewy body disease, frontotemporal lobar degeneration and motor neuron disease. In a subset of American National Football League players who had sustained concussions while playing found that CTE was associated with increased duration of football play and age at time of death. This observation suggests incubation and interactive effect of the prior injury with time and aging effects. McKee et al. (2012) conclude that the association of CTE “… with other neurodegenerative disorders suggests that repetitive brain trauma and hyperphosphorylated tau protein deposition promote the accumulation of other abnormally aggregated proteins including TAR DNA-binding protein 43, amyloid beta protein and alpha-synuclein (p. 43).” Dementia pugilistica has been diagnosed in as high as 20% of retired boxers and may have its onset long after the last boxing match (Kokjohn et al., 2013).

Lehman et al. (2012) found in retired National Football League (NFL) players, a rate of death associated with a neurodegenerative disorder 3 times higher than the general U.S. population. Hart et al. (2013) have also shown in aging, retired NFL players that WM pathology in DTI analyses is related to cognitive dysfunction and depression. While the Plassman et al. (2000) study (which verified presence of TBI in the medical record) reported a positive relationship between prior brain injury and development of dementia, some studies that include self-report of prior TBI do not (Dams-O'Connor et al., 2012). The issue is likely complicated because in the Plassman et al. study, when the mild TBI subjects were assessed independently, as a group they did not have a significantly increased hazard ratio for developing dementia. Returning to the model offered by Jack et al. (2013) and shown in Figure 8, those within the mild end of the TBI spectrum with a single head injury would likely have the least risk. Since mTBI constitutes the majority of those with brain injury, the high presence of mTBI with potentially minimal risk factors may be an explanation why some studies do not find an association. Using a retrospective cohort design with documented TBI, Wang et al. (2012) did demonstrate an increased risk for dementia. Sayed et al. (2013) examined a large cohort of individuals with TBI from the National Alzheimer's Coordinating Center Uniform Data set and observed that those with chronic cognitive deficit were those who met criteria for developing dementia. The Sayed et al. study may show that TBI alone may be insufficient to develop dementia, but TBI plus risk factors as outlined in Figure 9, reproduced from Smith (2013), such as residual cognitive impairment may be the combination needed for the transition from recovered but not demented to developing dementia post-injury.

Currently, all of the degenerative disorders including CTE require post-mortem confirmation, but ante-mortem neuroimaging studies may provide important insights into how brain injury interacts with the aging process and the development of late neuropathological sequelae. Koerte et al. (2012a,b) have shown DTI changes in athletes, even without symptomatic concussion. What it means to have abnormal DTI findings in the athlete with a history of concussive or sub-concussive blows to the head is not known at this time, but raises the specter of potential later-in-life reduced brain reserve capacity and increased vulnerability for neuropsychiatric disorder (Bigler et al., 2013).

Recently, Bailey et al. (2012) demonstrated in relatively young professional boxers (~28 years of age) that impaired cerebral hemodynamic function related to history and intensity of sparring and reduced neuropsychological performance. More than three decades after their last concussion, Tremblay et al. (2013) examined athletes with a history of prior concussion compared to those without. Athletes with prior concussions had abnormally enlarged ventricular size, cortical thinning in regions more vulnerable to the aging process and diminished episodic memory and verbal fluency compared to age matched athletes without prior history of concussion, yet demographically matched. Gardner et al. (2012) review the literature on sport-related concussions and DTI showing residuals in terms of WM damage even in this mild end of the TBI spectrum. In a within-subject prospective design, Toth et al. (2013) have shown a statistically significant subtle loss of brain volume by 1 month post injury in mTBI, where TBV was reduced by 1% and ventricular volume increased by 3.4%. However, without pre-injury baseline imaging, what role inflammation may have played in this is not known. These observations indicate that even mild injuries have the potential to initiate a cascade of neuropathological events that influence ageing and the potential to develop a neurodegenerative disorder. Complex genetic (Toth et al., 2013) factors are also likely related to how even mild injury influences outcome and the risk for late in life dementia (Ponsford et al., 2011; Schipper, 2011).

Finally, neuroinflammation specific to WM may play a role in the progression of degenerative brain changes for months to years' post-injury (Smith et al., 2012; Bigler, 2013; Johnson et al., 2013). There may be differential effects of WM inflammatory reactions between child and adult brain injury (Mayer et al., 2012) and DTI may provide a method for *in vivo* tracking of these changes (Voelbel et al., 2012).

## Summary

Directly related to the effects of trauma and its severity, TBI may induce widespread neuronal loss and disrupted WM connectivity as part of the primary and secondary effects of the injury along with injury-initiated neuroinflammation and neurodegeneration. Thus, TBI, especially toward the severe end of the spectrum, becomes a major risk factor for untoward effects later in life by reducing brain reserve capacities and diminished neuroplasticity to offset age-mediated decline. In fact, TBI may initiate an adverse synergistic effect with aging to predispose the earlier development of neuropsychiatric symptoms and age-related neurodegenerative diseases in the brain injured individual.

### Conflict of interest statement

Dr. Bigler co-directs a neuropsychological assessment lab wherein expert testimony in cases of traumatic brain injury may be given.
